# Evidence of High-Risk Human Papillomavirus in Esophageal Cancer in East Azerbaijan Province, Northwest of Iran

**DOI:** 10.1155/2022/1099477

**Published:** 2022-01-07

**Authors:** Zahra Sadeghian, Hossein Bannazadeh Baghi, Vahdat Poortahmasebi, Javid Sadeghi, Alka Hasani, Arezoo Azadi, Mahin Ahangar Oskouee

**Affiliations:** ^1^Department of Microbiology and Virology, School of Medicine, Tabriz University of Medical Sciences, Tabriz, Iran; ^2^Infectious and Tropical Diseases Research Center, Department of Microbiology and Virology, School of Medicine, Tabriz University of Medical Sciences, Tabriz, Iran

## Abstract

**Background:**

Human papillomavirus (HPV) is one of the most important viral agents associated with several classes of cancers in humans. The aim of this study was to investigate HPV in esophageal cancer in the East Azerbaijan province, northwest of Iran.

**Methods:**

140 paraffin-embedded specimens of esophageal tissues were investigated using nested-polymerase chain reaction (nested-PCR) with primer designing for the L1 region of HPV genome. According to the pathological diagnosis, the samples were divided into two groups: 70 patients with esophageal cancer EADC (*n* = 35) and ESCC (*n* = 35) as the case group and those without tumour in esophagus tissue as a control (*n* = 70).

**Results:**

HPV DNA was isolated from 20 (28.57%) of the 70 paraffin-embedded tissue specimens of esophagus cancer. Of these, 6 cases (17.14%) of EADC and 14 cases (40%) of ESCC were positive. In contrast, all cases of the control group were negative for the HPV genome. Sequence analysis revealed that HPV types 16 and 18 are the most frequent ones identified in this study.

**Conclusion:**

The prevalence of HPV in esophageal cancer can vary depending on the geographical location and other factors. Based on the findings of this study, HPV infection may possibly have contributed to an increased risk of esophageal cancer in a group of patients in Tabriz.

## 1. Introduction

The esophageal carcinoma is the sixth leading cause of cancer death and also the seventh most common cancer in the world [1]. Esophageal squamous cell carcinoma (ESCC) and adenocarcinoma (EADC) are two major histologic types of esophageal cancer with varying epidemiology, primary risk factors, and time trends [2]. EADC is most commonly found in the lower third of the esophagus, whereas ESCC mostly affects the squamous cells located in the upper two-thirds of the esophagus [3]. Countries such as China, South Africa, Iran, Brazil, France, and Singapore are considered as high incidence areas for esophageal cancer [4]. Directly or indirectly, viral infections can play a role in various stages of carcinogenesis, and development of malignant tumours and human papillomavirus is one of the important viral agents that can play a role in carcinogenesis [5]. Syrjänen et al. in 1982 presented the first report of HPV in esophageal cancer [6]. Several studies have shown the prevalence rates of HPV in esophagus cancer between 0% and 71%. Differences in the results of the studies are probably due to differences in samples, geography, and the sensitivity and specificity of the techniques used [7, 8]. The aim of this study was to investigate HPV in esophageal cancer in East Azerbaijan province, northwest of Iran.

## 2. Materials and Methods

### 2.1. Study Population

In the present study, 140 paraffin-embedded specimens of esophageal tissues were collected from the Pathology Department of Imam Reza Hospital in Tabriz (2017–2019). Of these, 70 samples were related to patients with esophageal cancer (as the case group) and 70 samples to noncancerous patients (as the control group), both of which were diagnosed and confirmed by the pathologist.

The obtained samples were transferred under appropriate conditions to Tabriz University of Medical Sciences. Medical and demographic information relevant to each patient were obtained from archived medical records of the pathology department. The age range of patients with esophageal cancer and the control group was (26–98) and (28–97) years, respectively.

### 2.2. Sample Preparation and DNA Extraction

Thin sections were prepared from paraffin-embedded esophageal tissue specimens. To prevent cross-contamination between samples, new scalpel razors and disposable gloves were used for each sample. Additionally, all steps were performed under the biological hood. Then, the samples were collected in a sterile microtube. Subsequently, sections were deparaffinized by xylene and subsequently rinsed using ethanol. After ensuring complete evaporation of the ethanol from the samples, DNA extraction was carried out according to Yekta-Tajhiz kit protocol (DNA extraction from the paraffin tissue kit, Taiwan).

To ensure the quality and control of the extracted DNA and rejection of the presence of PCR inhibitors, the OD of the samples was read using a spectrophotometer, and then, the PCR test was performed on all samples for *β*-globin gene [9].

#### 2.2.1. Nested-Polymerase Chain Reaction Assay (Nested-PCR)

After confirming the quality of the extracted DNA, the HPV genome was detected by nested-PCR assay. The first stage of nested-PCR was performed using MY09/MY11 primer pairs that amplified the L1 gene [9]. These primers traced a wide range of HPV types, and the PCR product size was 450 bp. The ampliﬁcation mixture consisted of 10x PCR buffer, Mgcl2 (25 mM), dNTps (10 mM each), Taq DNA polymerase (5 u/*µ*l), primer MY09 (10 *µ*M), primer MY11 (10 *µ*M), and 500 ng of DNA template in a ﬁnal volume of 50 *µ*l. At first, DNA was denatured for 5 min at 94°C, followed by 38 amplification cycles at 94°C for one minute, 55°C for one minute, and 72°C for one minute, and the ﬁnal extension step was 7 minutes at 72°C.

The GP5+/GP6+ primer pairs were used in the second step of nested-PCR. The ampliﬁcation mixture consisted of 10x PCR buffer, Mgcl2 (25 mM), dNTps (10 mM each), Taq DNA polymerase (5 u/*µ*l), primer GP5+ (10 *µ*M), primer GP6+ (10 *µ*M), and 500 ng of DNA template in a ﬁnal volume of 50 *µ*l. At first, DNA was denatured for 5 min at 94°C, followed by 38 amplification cycles at 94°C for one minute, 40°C for 1.5 minute, and 72°C for 1.5 minute, and the ﬁnal extension step was 7 minutes at 72°C. In both PCR reactions, extracted DNA from HeLa cell line were used as positive control, and distilled water was used as negative control. The PCR product at this stage was 150 bp.

### 2.3. Gel Electrophoresis

The PCR products were evaluated by 1.5% agarose gel electrophoresis. Then, the results of electrophoresis were observed under UV light by the gel documentation system. Following, positive PCR products were subjected to DNA sequence analysis.

### 2.4. HPV Typing and Phylogeny

Direct sequencing of L1 genes was carried out (Perkin Elmer ABI-3130XL DNA Sequencer, Foster City, CA, USA) using 0.5 *μ*L of appropriate primers GP5+ and GP6+ for surface gene. The electropherograms were examined visually using the Chromas software. Sequences of the L1 genes were aligned using the BioEdit software (version 7.0.9).

Genotyping was performed by phylogenetic analysis with reference sequences of HPV genotypes. Phylogenetic tree generation was conducted using the MEGA software (version X), with the maximum likelihood (ML) method in the Kimura two-parameter substitution matrix B [10]. Bootstrap resampling and reconstruction were carried out 1000 times to confirm the reliability of the phylogenetic tree [11]. Significance was based on bootstrap values of 70. The L1 gene sequence of the bovine papillomavirus (BPV) was used as an out-group.

### 2.5. Statistical Analysis

The data analyses were conducted using SPSS software (version 25). Descriptive statistical tools such as mean and standard deviation were used to display quantitative data. Frequency and percentage were used to display qualitative data. The independent *t*-test was performed to compare the mean age between case and control groups. Furthermore, the chi-square test was used to compare sex, age groups, and pathology sample for HPV between case and control groups. *P* values of less than 0.05 were considered as statistically significant.

## 3. Results

In the current study, 150 paraffin-embedded esophagus tissue blocks were collected. The quality of the extracted DNA was found unsuitable in 10 samples; therefore, they were excluded from the study. Subsequently, we investigated 140 paraffin-embedded tissue blocks that were investigated in two groups based on the pathological diagnosis: 70 patients with esophageal cancer EADC (*n* = 35) and ESCC (*n* = 35) as case and those without esophageal tumour as control (*n* = 70). In general, the HPV genome was found in 20 (28.57%) out of 70 paraffin-embedded tissue specimens of esophagus cancer (case group). Six cases (17.14%) of EADC and 14 cases (40%) of ESCC were positive for the presence of HPV genome. Nevertheless, none of the control samples was positive for HPV DVA. The clinical characteristics of the patients are given in Table 1. The positive PCR products were subjected to DNA sequencing analysis. The clinical data of 20 HPV-positive patients are given in Table 2. The results of HPV sequencing revealed the presence of HPV-16 in EADC 20% (4/20) and in ESCC 25% (−5/20) and HPV-18 in EADC 10% (2/20) and in ESCC 45% (9/20). The phylogenetic tree was based on L1 sequencing constructed using MEGA software (version X), with the maximum likelihood (ML) method in the Kimura two-parameter substitution matrix B.

## 4. Discussion

Cancer is the second leading cause of death in the world behind cardiovascular disease [12]. Due to poor prognosis and rapid progression, esophageal cancer is one of the most important gastrointestinal cancers with seventh place in terms of cancer occurrence in the world. The esophageal cancer is the fifth most common cancer in developing countries [13, 14]. According to the report of the International Agency for Research on Cancer (IARC) in 2012, annually, about 90% of the 456,000 cases of esophageal cancer are related to ESCC [15–17]. ESCC is more prevalent in developing countries and affects most of the squamous cells present in the upper esophagus. Whereas, EADC is commonly found in cells present in the lower third of the esophagus, and its prevalence is higher in developed countries.

Since 1982, when the first report of communication between HPV infection and esophageal cancer was published, many studies have been carried out in this area [18].

In the present case-control study, the HPV DNA L1 region was examined in both types of esophageal cancer (EADC and ESCC) in the case and control groups. The results showed that 28.57% of all esophageal cancer cases were positive for the HPV L1 gene. In esophageal adenocarcinoma, six cases (17.14%) and in esophageal squamous cell carcinoma, 14 cases (40%) were positive for the HPV genome. No positive samples were observed in the control group.

In China, based on the National Central Cancer Registry report, the fourth leading cause of death from cancer was related to esophageal cancer. The prevalence of HPV in the ESCC varied from 6.7% to 83.3% [19, 20].

In a study in the high-risk area of South Africa (Transkei), the prevalence of HPV in esophageal cancer samples was estimated to be 46% [7]. In Turkey, 63.3% and 40% of patients with ESCC and EADC were positive for HPV DNA, respectively, using the PCR test [21].

According to the reports from the International Agency for Research on Cancer (IARC) in 2018, age-standardized incidence rate (ASR) per 100,000 for esophagus cancer in Iran in both males and females was 6.6 and 5.2, respectively. Additionally, age-standardized mortality rate (ASMR) per 100,000 for esophagus cancer in Iran was 2.9 [22]. The prevalence of esophageal cancer in Iran is also variable in different regions.

In a study in the high-risk area of Iran (Turkmen Sahra), HPV DNA was recognized in 49.4% of patients with ESCC, and the positive cases encompassed HPV-16 (54.7%), HPV-18 (4.8%), HPV-6 (14.3%), HPV-66 (7.1%), and HPV-52 (4.8%) [23].

In a case-control study in Iran (Tehran), the HPV L1 gene was detected in 36.8% of ESCC samples and 13.2% of control samples. Among positive samples, HPV-16 and HPV-18 were observed using type-specific E6/E7 primers in 13.2% and 7.9% of ESCC samples, respectively. While, 13.2% of the control samples were positive for the HPV-18 E6/E7 gene [24].

In another study in Mazandaran, Northern Iran, 177 cases of ESCC were investigated. Of these, 49 cases (27.68%) were positive for HPV DNA [8].

In the present study, the prevalence of HPV in EADC (17.14%) and in ESCC (40.0%) was lower than previously conducted studies in the high-risk areas for ESCC. Therefore, based on the results of the chi-square test, significant differences in the prevalence of HPV were observed in the case group than in the control group (*P* < 0.0001). Nevertheless, the values obtained in this study were lower than the high-frequency areas elsewhere in the world and in Iran. Differences in sample size, research design, the sensitivity of the diagnostic methods, statistical analysis, environmental factors contributing to esophageal cancer incidence, nutritional habits, and race may be responsible for the differences in the results of the studies in various regions.

In general, almost 20% of cancers have viral origins. Human papillomavirus is one of the viral factors that is likely associated with esophageal cancer [4, 25].

HPVs are divided into low-risk and high-risk variants. Low-risk types usually lead to benign lesions, while high-risk types can cause carcinogenesis by interfering with the pathways regulating cell proliferation [26]. High-risk HPV strains are responsible for approximately 5% of human cancers [27]. Nonetheless, low-risk types of HPV (HPV-6 and HPV-11) have been reported in some cancers [28].

In the present investigation, HPV type 18 and type 16 were detected. In ESCC samples HPV-18 with 64.28% and in EADC samples HPV-16 with 66.6% were the predominant types.

Evidence obtained from several studies suggest a correlation between high-risk HPV genotypes (mostly 16 or/and 18) and esophageal cancer [8, 21, 29–32].

In this study, the high-risk genotypes of 18 and 16 were detected in HPV-positive samples by analysis of sequencing, as reported in most other high-risk areas.

## 5. Conclusions

The results of this study revealed significant differences in prevalence of HPV between the case group and control group which is in consistent with previous studies. Another finding was that the presence of high-risk genotypes (16 and 18) implicates HPV as one of the possible etiological factors in esophagus cancer. As different rates of prevalence of HPV in esophageal cancer have been reported from Iran and other parts of the world, a further extensive investigation with larger numbers of subjects over wider geographical areas is needed to strongly support the role of human papillomavirus in esophageal cancer.

## Figures and Tables

**Table 1:** The description of gender, age, and HPV positivity in case and control samples.

	Cancer samples (case)			Noncancerous samples (control)	*P* value
	Total, *N* = 70	EADC, *N* = 35	ESCC, *N* = 35	Total, *N* = 70	
Gender					
Female	31 (44.28%)	15 (42.85%)	16 (45.71%)	34 (48.57%)	0.611
Male	39 (55.71%)	20 (57.14%)	19 (54.28%)	36 (51.42%)	

Age, years					
≤50	41 (58.57%)	22 (62.85%)	19 (54.28%)	30 (42.85%)	0.063
>50	29 (41.42%)	13 (37.14%)	16 (45.71%)	40 (57.14%)	
Mean age	51 ± 17.8	49.97 ± 15.11	52.08 ± 20.45	56.77 ± 18	

HPV					
+	20 (28.57%)	6 (17.14%)	14 (40.0%)	0 (0.00%)	0.0001
_	50 (71.42%)	29 (82.85%)	21 (60.0%)	70 (100.0%)	

HPV, human papillomavirus; ESCC, esophageal squamous cell carcinoma; EADC, esophageal adenocarcinoma.

**Table 2:** Correlation between the presence of HPV subtype and the clinical and pathological characteristics in HPV-positive samples (*N* = 20).

Pathological characteristics of esophagus cancer, *N* (%)	Age average (year)	Gender, *N* (%)	HPV-16 (+), *N* (%)	HPV-18 (+), *N* (%)
EADC 6 (30%)	55.6	M 3 (50%)	2 (66.66%)	1 (33.33%)
		F 3 (50%)	2 (66.66%)	1 (33.33%)

ESCC 14 (70%)	47.28	M 6 (42.85%)	1 (16.66%)	5 (83.33%)
		F 8 (57.14%)	4 (50%)	4 (50%)

HPV, human papillomavirus; ESCC, esophageal squamous cell carcinoma; EADC, esophageal adenocarcinoma; F, female; M, male.
